# The RonchAP® palatinal device: A conservative approach in treating obstructive sleep apnea syndrome—a randomized, controlled study

**DOI:** 10.1007/s00405-022-07738-4

**Published:** 2022-11-28

**Authors:** Sophie Lembacher, Sophia Gantner, Bernd Uhl, Martin Holzer, Martin Patscheider, John-Martin Hempel

**Affiliations:** 1 University Clinic of Dentistry, Medical University of Vienna, Sensengasse 2a, 1090 Vienna, Austria; 2 Department of Otorhinolaryngology, Head and Neck Surgery, Ludwig Maximilian University Munich, Munich, Germany; 3 Department of Otorhinolaryngology, Head and Neck Surgery, Südtiroler Sanitätsbetrieb, Brixen, Italy

**Keywords:** Palatal device, Obstructive sleep apnea syndrome, Ronch®AP, Snoring, Conservative treatment

## Abstract

**Purpose:**

The aim of the present study was to assess the efficacy of the Ronch®AP palatal device in treating patients with moderate and severe forms of obstructive sleep apnea syndrome.

**Methods:**

In a randomized controlled trial 22 patients were examined with the Ronch®AP palatal device after 4 weeks of usage. Their results were compared to a control group of 30 patients who did not receive any treatment during this time. All patients included did not tolerate CPAP therapy. Among other parameters the apnea–hypopnea index (AHI) was measured using nocturnal cardiorespiratory polysomnography. Daytime sleepiness was assessed using Epworth Sleepiness Scale. Pittsburgh Sleep Quality Index was used to analyze sleep quality.

**Results:**

Using the Ronch®AP palatal device AHI was reduced from an average of 35.34 ± 14.9/h to 19.18 ± 14.93/h, whereas the control group only showed a minimal mean reduction from 31.32 ± 12.76/h to 29.37 ± 17.11/h. The difference in reduction between the two randomized groups was highly significant (*d* = − 14.2, 95% CI 5.9–22.6, *t* = 3.4, *df* = 49.9, *p* = 0.001). Epworth Sleepiness Scale score was lowered from 9.18 ± 4.73 to 7.82 ± 4.14 on average and sleep quality improved by − 1.91 ± 2.31. Both changes were also statistically relevant (*p* < 0.005).

**Conclusions:**

The Ronch®AP device is an effective alternative treatment option for patients suffering from moderate and severe forms of obstructive sleep apnea syndrome and not tolerating CPAP therapy.

**Trial registration number:**

407-16 with approval from the local ethical committee (Ethikkommission der Medizinischen Fakultät der LMU München).

## Introduction

Obstructive sleep apnea syndrome (OSAS) is a common chronic disorder caused by repetitive pharyngeal collapse during sleep leading to complete or partial airway obstruction during inspiratory airflow. It is characterized by frequent reoccurrences of hypopneas and apneas resulting in strongly compromised blood oxygenation levels resulting in frequent arousals and disrupted sleep often causing excessive daytime sleepiness [1]. There is strong evidence recognizing that OSAS is often associated with severe complications including hypertension, coronary artery disease, arrhythmias, heart failure, stroke, metabolic syndrome, diabetes and neurocognitive disorders [2–4]. Patients frequently complain about cognitive impairments, such as changes in attention span and concentration capacity [4]. The health complications mentioned emphasize the importance of early diagnosis and therapeutic intervention. As the number of patients suffering from OSAS is expected to increase in the future due to the increasing prevalence of obesity, a major risk fact for OSAS, it represents an important public-health problem [4, 5].

OSAS diagnosis is based on the combined evaluation of clinical symptoms and objective sleep study findings. Cardinal symptoms include loud snoring or choking, frequent awakenings and excessive sleepiness. The gold standard to confirm the clinical suspicion of OSAS is cardiorespiratory polysomnography with its primary diagnostic parameter being the apnea–hypopnea index (AHI). Apnea is defined as the complete absence of inspiratory airflow, whereas during hypopnea inspiratory airflow is only reduced. OSAS is defined as five or more episodes of apnea or hypopnea per hour of sleep in association with clinical symptoms (e.g., excessive daytime sleepiness, fatigue, impaired cognition or cardiovascular events) or 15 or more obstructive apnea–hypopnea events per hour of sleep regardless of any associated symptoms. Polysomnography is not only limited to OSAS diagnosis. It also proves to be a useful instrument in assessing severity and guiding therapeutic intervention [6]. Furthermore, drug-induced sleep endoscopy (DISE) can be used to document a localization of obstructions and contribute to the selection of an individualized and appropriate therapeutic intervention based on these results. Outcomes of surgery appears to be improved by the localization of the site of collapse by DISE [7].

The therapeutic spectrum includes behavioral, conservative and surgical treatment options. Continuous positive airway pressure (CPAP) represents the treatment of choice in most patients suffering from OSAS [8]. It has been effective in reducing symptoms, cardiovascular and neurocognitive sequelae as well as mortality [9–11]. Due to the chronic nature of the disease, a long-term treatment approach with high compliance levels is essential in reducing the risk of severe complications. A major limitation in CPAP therapy is, however, its high variability in therapy acceptance and therapy adherence. Despite its high effectiveness, tolerance rates between 29% and 83% are rather poor [12]. Because further technical improvements, since the introduction of CPAP therapy has not lead to increased compliance levels, the necessity for alternative treatment options ranging from conservative to surgical approaches is apparent [13].

In the latter, for example, there is the possibility of Barbed reposition pharyngoplasty or Expansion Sphincter Pharyngoplasty. In case of palatal collaps the outcomes in both procedures appear to be comparable in the improvement of OSA and are considered safe procedures [14, 15].

Conservative treatment approaches include oral appliances, such as mandibular protrusion splints and palatal stenting devices. While the effectiveness of mandibular protrusion splints has been thoroughly examined in recent years, scientific evidence for palatal stenting devices is rather scarce [11, 16]. So far, no scientific standards in regard to its functional construction have been formulated. Moreover, the German Sleep Society (DGSM) has not commented on the usage of palatal stenting devices for OSAS treatment in its latest guideline on sleep-related breathing disorders [17].

This study presents the first controlled and randomized clinical trial to examine the effectiveness of the Ronch®AP palatal device in treating OSAS patients with CPAP intolerance.

## Materials and methods

### Patients and features

**Fig. 1:**
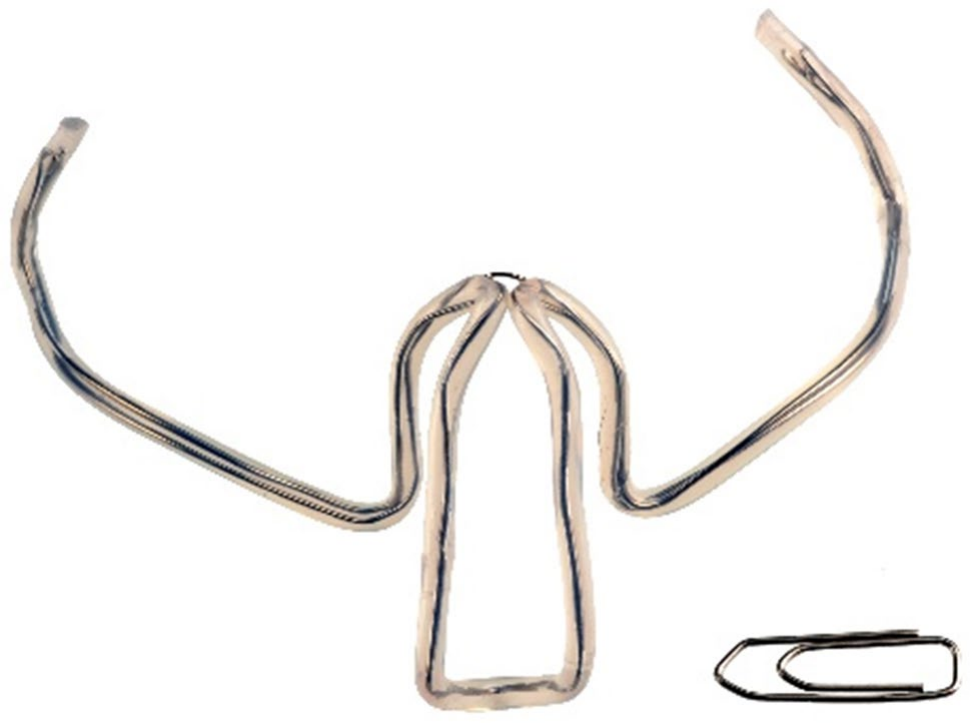
Ronch®AP palatal device: relative size to a paper clip. Source: RonchoLine®

**Fig. 2:**
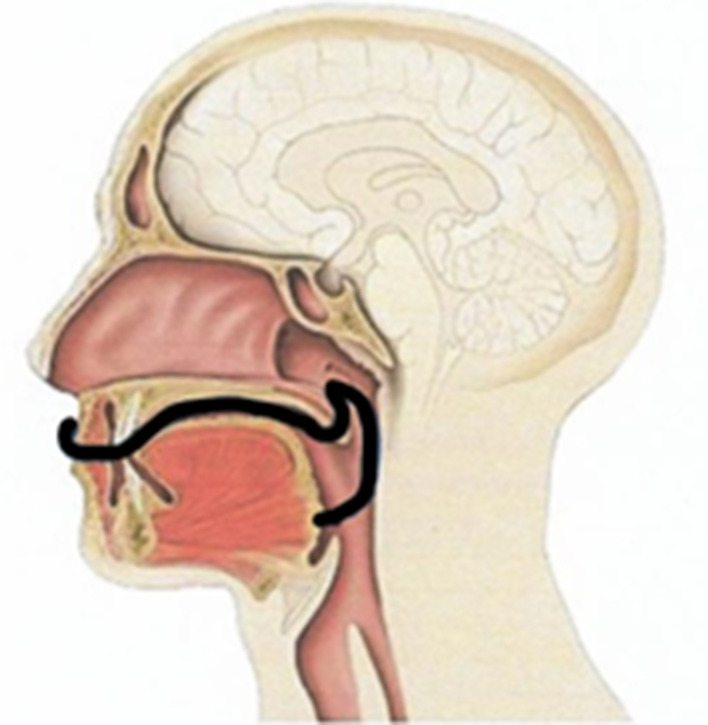
Positioning of Ronch®AP palatal device. Source: RonchoLine®

From October 2016 to August 2019, 60 patients were evaluated at the Department of Otorhinolaryngology of the Ludwig-Maximilians-University Clinic in Munich. The study examining the effectiveness of the Ronch®AP palatal device in treating OSAS was designed as a randomized and controlled trial. The functional principle of the Ronch®AP is based on the external stenting of the retropalatal space and follows a conservative treatment approach. It is based on the nocturnal usage of an individually adjusted plasticized wire construction with memory effect (Fig. 1). The device is applied through the mouth and advances the velum, the tongue base and the posterior pharyngeal wall by stenting the retropalatal space prone to collapse (Fig. 2). The device is held and fixed by the lips through two retentive extensions. In contrast to mandibular protrusion splints, retention is not dependent on dental status. Behind the lips, the wire lies laterally on both sides of the alveolar process and crosses medially in the retromolar region. Then, it continues to the retropalatal space in the form of two slightly anteriorly benched bows. The first bow lies behind the velum, thus advancing and stenting it. A second bow extends into the naso- and oropharnyx, thus stenting the posterior pharyngeal wall. A ventrocaudal appendix, forming as an extension of the second bow, stabilizes the tongue base. The freely titratable inflections allow individual adjustment in accordance with the patient’s anatomy.

Only patients suffering from moderate to severe OSAS with an AHI ≥ 15, and who did not tolerate CPAP therapy, were included. Moderate OSAS was defined as AHI ≥ 15/h, severe OSAS as AHI ≥ 30/h. All patients went through a diligent ENT examination including rhinoscopy, pharyngoscopy and inspection of the oral cavity. If OSAS had previously not been formally diagnosed, but was assumed based on the results of clinical screening and analysis of risk factors, standardized cardiorespiratory polysomnography was conducted for verification. Polysomnographic testing was conducted at the sleep laboratory of the Department of Otorhinolaryngology of the Ludwig-Maximilians-University Clinic in Munich in accordance with AASM guidelines [1]. Further inclusion criteria comprised a body-mass index (BMI) under 32 and NYHA class I. Patients suffering primarily from central apnea, hypertrophy of tonsils, malignant forms of cancer and cardiac insufficiency (NYHA class II–IV) were excluded from participating. Furthermore, pregnant women and patients under the age of 18 were not eligible for study participation and were excluded in advance.

In addition to polysomnographic parameters daytime sleepiness was assessed using Epworth Sleepiness Scale (ESS) [18, 19]. Pittsburgh Sleep Quality Index (PSQI) was used to measure sleep quality. The index considers subjective sleep quality, sleep latency, sleep duration, habitual sleep efficiency, sleep disturbances, use of sleeping medication and daytime dysfunction. The sum of these subscores for these seven components yields one global score [20]. Both questionnaires are validated instruments in sleep medicine facilitating the evaluation of subjective treatment success. Low ESS scores indicate little sleepiness. Low PSQI scores point to good sleep quality. Both questionnaires were evaluated after the patient’s registration and consent for study participation.

### Randomization

Afterward, patients were randomized into one group set to receive therapeutic intervention by Ronch®AP and a control group. For randomization an envelope containing equal amounts of batches for both groups was used. Each group consisted of 30 patients.

### Treatment protocol and follow-up

After randomization, the Ronchr®AP palatal device was individually adjusted in 30 patients by a cooperating dental laboratory. Dental technicans had previously been teached by the inventors of the Ronch®AP device. In total, there were three dental technicans who chaperoned and monitored the adaptation process.

**Fig. 3:**
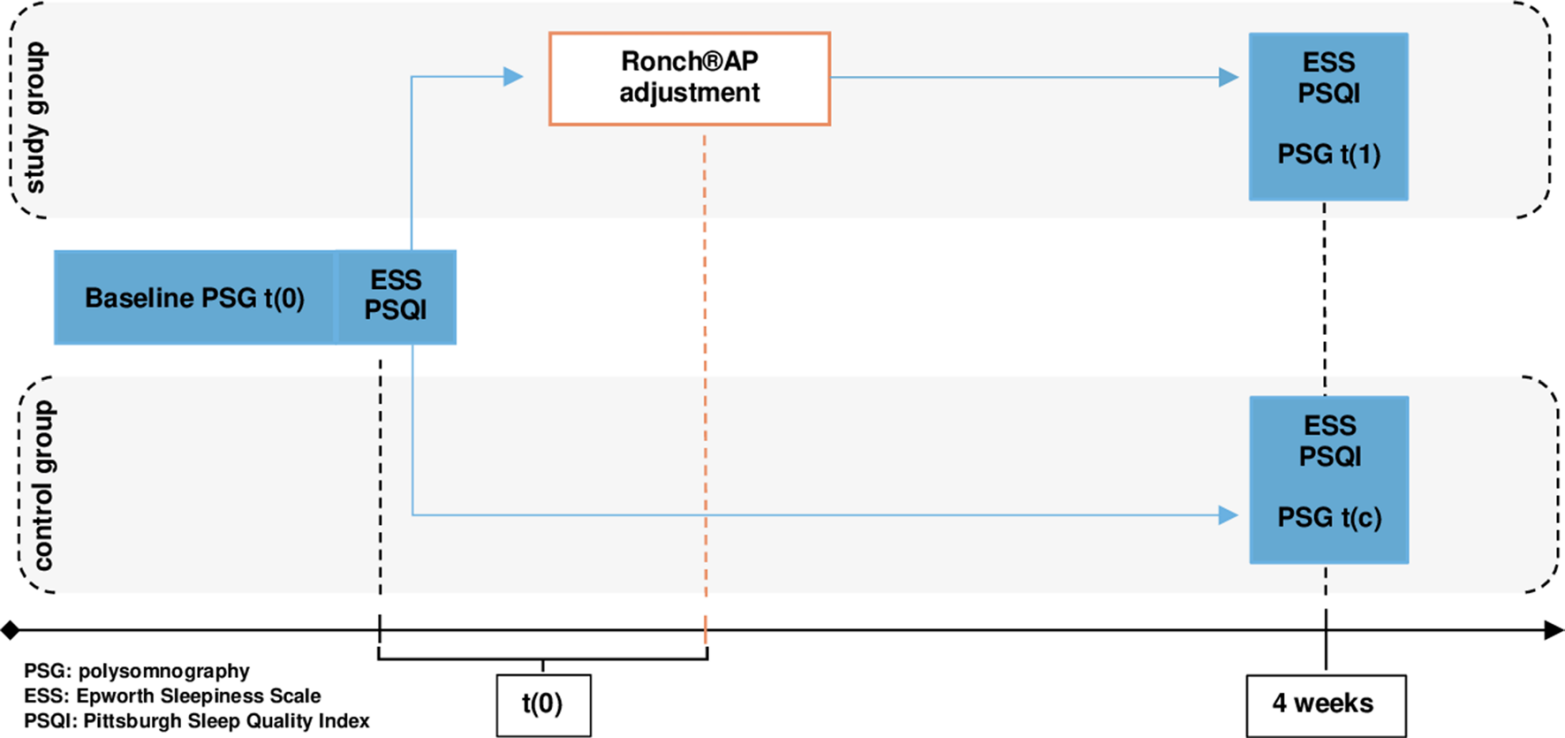
Consort flow diagram

After 4 weeks patients of both groups were tested using 16-canal cardiorespiratory polysomnography at the interdisciplinary sleep laboratory at the Ludwig-Maximilians-University Clinic (Fig. 3). Again polysomnographic testing was conducted in accordance with international standards. At the beginning of polysomnography patients were calibrated under supervision of qualified sleep laboratory personnel, while they were still awake to facilitate the differentiation of physiological sleep functions and artefacts. Collected data were analyzed using an automatic analyzing software and consecutively manually reviewed by trained sleep laboratory personnel. ESS and PSQI were evaluated simultaneously in the morning after polysomnographic testing. Table 1 gives an overview of all parameters that were extracted from polysomnographic report and evaluated.

**Table 1:** Overview on primary and secondary parameters used for evaluation of RonchAP® effectiveness

Primary parameter	Secondary parameters
Apnea–hypopnea index (AHI)	**Polysomnographic parameters:** • Apnea index (AI) and hypopnea index (HI) • Arousal-reaction due to increased respiratory work (respiratory effort-related arousal, RERA) • Average oxygen saturation (SpO2 Ǿ) • Minimum oxygen saturation (SpO2 minimal) • Snoring index and total amount of snoring sounds • Sleep stages REM, N1, N2, N3 • Wake after sleep onset (WASO) **Results of the sleep questionnaires:** • Epworth Sleepiness Scale (ESS) • Pittsburgh sleep quality index (PSQI) • Ronch®AP questionnaire

As AHI was defined to be the primary parameter, different criteria for treatment success were formulated in dependence with AHI. They were defined as follows: (1) AHI < 5/h: the definition of OSAS does not apply anymore; (2) AHI < 10/h: improvement of OSAS to a mild manifestation; (3) fulfillment of Sher criteria, that is an AHI < 20/under treatment and a 50% reduction with respect to baseline AHI.

To gain a more comprehensive understanding of subjective evaluation of Ronch®AP and to analyze potential side effects a questionnaire was specially designed for this study. Patients were asked about the average time of usage in nights per week and the average duration of usage in hours per night. Furthermore, patients’ wearing comfort and willingness to continue Ronch®AP treatment in the future were measured on a scale from 1 to 5. A value of 1 indicated no desire for treatment continuation and very poor wearing comfort. A value of 5 equaled a very high willingness for treatment continuation and very high wearing comfort. Side effects were examined in an open question segment.

### Statistical analysis

Program R (Version 4.0.1, R Core Team, 2020) was applied for statistical evaluation. For descriptive analysis of parameters absolute values as well as margins were used. Mean value, standard deviation, minimum and maximum were calculated. Two sample *t* tests were used for comparison of means of both groups. For analyzing relevant predictors, Pearson’s correlation and linear regression models were used for metric variables. Two factor analysis of variance was used to analyze the effect of categorial parameters on AHI variation (e.g., BMI, severity of OSAS). Fisher’s exact test (chi square with Yates correction for small sample size) was used to analyze the influence of sleeping position on AHI variation and treatment success. The level of significance was defined as *p* < 0.05.

## Results

After the adjustment period was completed, 22 out of 30 patients tolerated Ronch®AP and were approved for polysomnographic testing. In accordance with the study’s protocol 30 patients of the control group did not receive any treatment. 14 women (23.3%) and 46 men (76.75) were included in the study. 32 patients suffered from moderate (AHI < 30/h) and 28 from severe OSAS (AHI ≥ 30/h). Patients suffering from mild OSAS could not be included. Mean age was 58.7 ± 10.9 years and mean body mass index (BMI) 26.8 ± 3.1 kg/m^2^. AHI was 32.4 ± 13.7/h on average. ESS-Score reached 8.8 ± 4.2, and PSQI was 7.7 ± 3.3. The comparison of both groups showed no significant differences in basic initial parameters (Table 2). However, with 33.3% (*n* = 10) more females were included in the control group than in the therapy group (13.3%, *n* = 4). Eight patients of the therapy group terminated their participation prematurely due to little or no tolerance. After 1 month of Ronch®AP therapy 73.3% of patients (*n* = 22) were still compliant.

**Table 2:** Comparability of baseline parameters after randomization

Variable	Mean value ± SD		*t* test/Fisher’s exakter test
	Control	Ronch®AP	
Age [years]	58.2 ± 11.5	59.3 ± 10.5	*t* = − 037, *df* = 57.6, *p* = 0.714
BMI [kg/m^2^]	26.7 ± 3.6	26.9 ± 2.6	*t* = − 0.22, *df* = 53.5, *p* = 0.824
Initial AHI [n/h]	31.3 ± 12.8	33.4 ± 14.7	*t* = − 0.59, *df* = 56.9, *p* = 0.559
Gender ratio (female/male)	10/20 (33.3%/66.7%)	4/26 (13.3%/86.7%)	Odds ratio = 0.3, 95% CI 0.1–1.3, *p* = 0.125

**Fig. 4:**
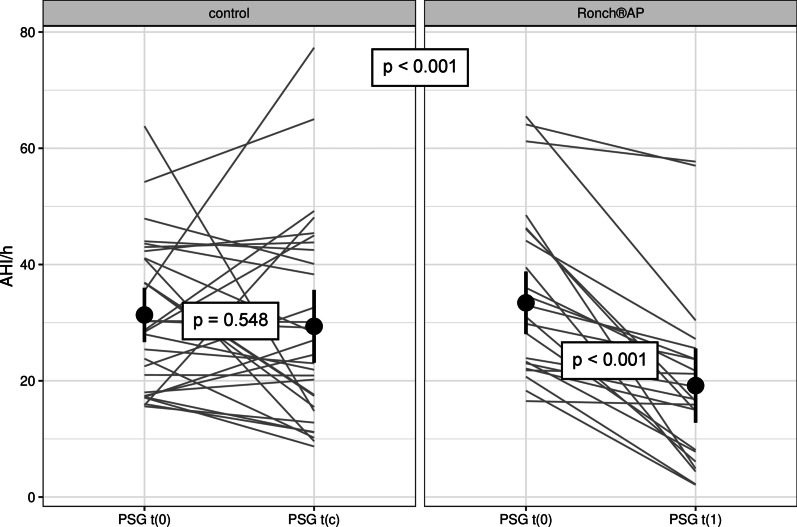
AHI

The effectiveness of the Ronch®AP palatal device was analyzed through the difference in parameter value between the two different times of measurement *t*(1) − *t*(0) or *t*(c) − *t*(0). *t*(0) was defined as baseline measurement for both groups, *t*(c) as follow-up of patients without treatment after 1 month and *t*(1) as follow-up of patients under Ronch®AP therapy. Negative differences implied that the parameter was reduced at the second time of measurement, suggesting an improvement. Positive differences implied an increase in parameter value over time, indicating a deterioration. 100% of patients treated with Ronch®AP showed negative differences in AHI, whereas in the control group, only 63% of patients had a lower AHI after 4 weeks. While the control group showed a mean AHI reduction from 31.32 ± 12.76/h to 29.37 ± 17.11/h (*d* = − 1.95 ± 17.59/h), Ronch®AP therapy improved AHI from 35.34 ± 14.9/h to 19.18 ± 14.93/h (*d* =  − 16.17 ± 12.40/h) (Table 3, Fig. 4). A two-sample *t* test confirmed a statistically significant difference in mean AHI reduction between both groups (*d* = − 14.2, 95% CI 5.9–22.6, *t* = 3.4, *df* = 49.9, *p* = 0.001).

**Table 3:** Effect of Ronch®AP therapy in comparison with no treatment: Baseline values and follow-up results after 1 month (absolute value)

Variable	Unit	Ronch®AP		Control	
		Initial (mean value + SD)	1 month follow-up (mean value + SD)	Initial (mean value + SD)	1 month follow-up (mean value + SD)
AHI	[n/h]	35.34 ± 14.9	19.17 ± 14.93	31.32 ± 12.76	29.37 ± 17.11
AI	[n/h]	12.3 ± 11.72	4.88 ± 11.25	8.77 ± 9.38	7.8 ± 10.89
HI	[n/h]	22.95 ± 10.33	15.06 ± 11.84	22.17 ± 11.79	20.73 ± 11.38
Apnea obstructive	[n]	61.35 ± 62.78	30.25 ± 78.49	46.15 ± 48.37	51.81 ± 74.79
Apnea central	[n]	1.25 ± 2.43	1.2 ± 3.44	8.26 ± 22.59	2.89 ± 8.85
Apnea total	[n]	62.8 ± 61.93	32.1 ± 81.26	56.22 ± 61.09	54.7 ± 78.03
Hypnea total	[n]	116.4 ± 50.71	87.64 ± 89.83	131.38 ± 71.08	129.42 ± 74.59
SpO2 Ǿ	[%]	93.31 ± 1.14	92.67 ± 1.8	93.44 ± 1.47	93.49 ± 1.33
SpO2 minimal	[%]	79.39 ± 8.22	81.42 ± 8.24	80.71 ± 8.41	80.07 ± 8.71
RERA	[n]	11.9 ± 41.06	0.29 ± 0.78	4.21 ± 10.65	3.29 ± 6.79
Sleep efficiency	[%]	72.81 ± 15.15	75.76 ± 16.82	79.95 ± 9.6	80.37 ± 8.86
N1	[%]	19.31 ± 12.53	15.77 ± 7.84	16.19 ± 10.23	16.48 ± 7.02
N2	[%]	56.09 ± 11.37	55.57 ± 9.86	57.4 ± 9.86	57.02 ± 8.63
N3	[%]	10.48 ± 6.54	10.61 ± 5.29	10.19 ± 5.67	8.94 ± 5.19
REM	[%]	14.13 ± 5.48	17.43 ± 6.83	15.81 ± 7.42	17.23 ± 6.11
WASO	[min]	81.92 ± 60.25	57.19 ± 45.47	64.07 ± 36.68	58.87 ± 36.22
Snoring index	[n/h]	188.47 ± 112.85	116.18 ± 116.27	248.84 ± 178.23	234.96 ± 140.45
Snoring sounds total	[n]	1043.94 ± 698.56	641.17 ± 658.2	1487 ± 1097.62	1451.85 ± 957.68
ESS	–	9.18 ± 4.73	7.82 ± 4.14	8.2 ± 4.17	8.83 ± 3.88
PSQI	–	8.45 ± 3.46	6.55 ± 3.46	7.27 ± 3.37	7.17 ± 3.13

**Fig. 5:**
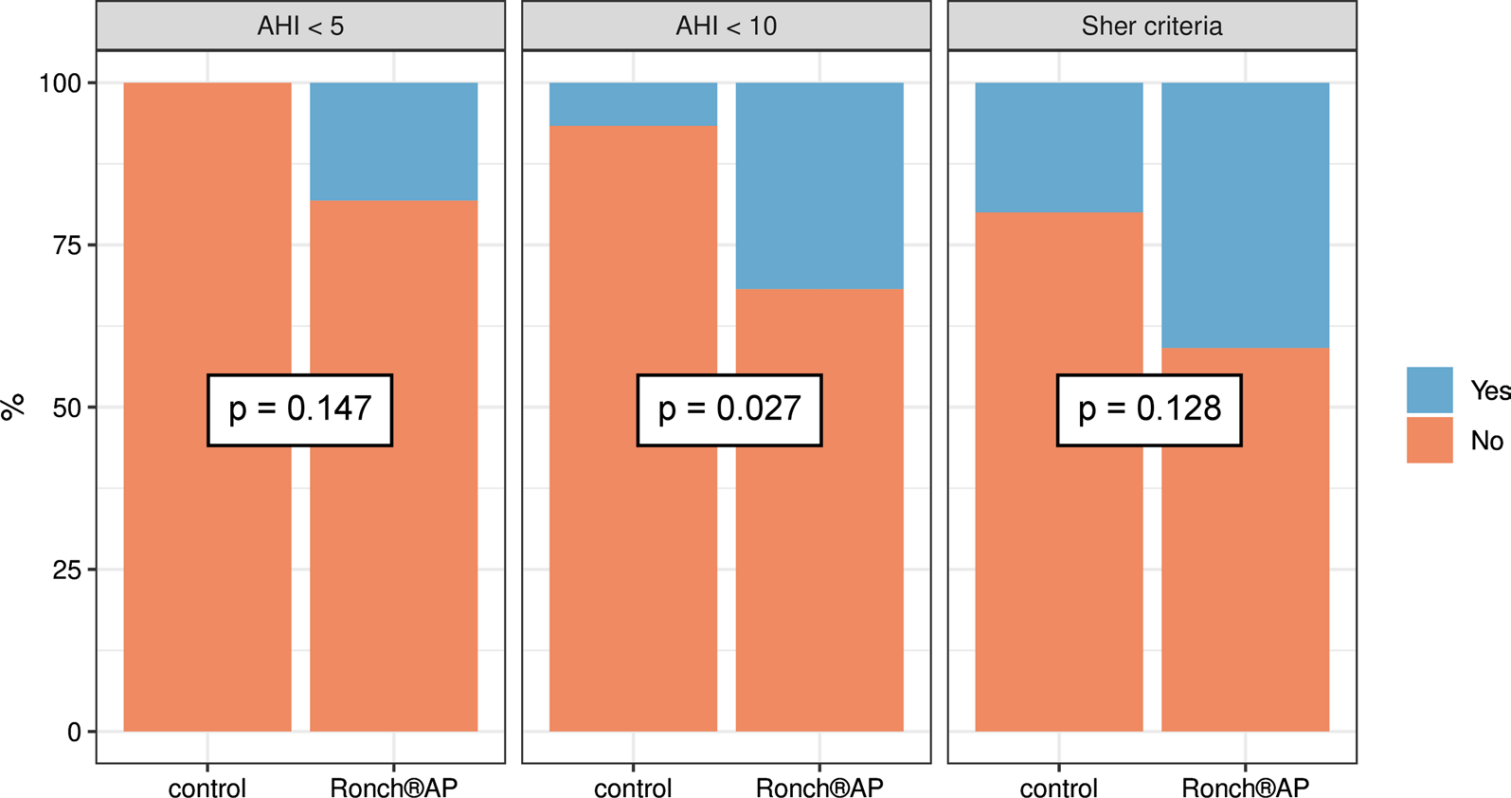
Treatment success in regard to AHI

Correspondingly, the analysis of treatment success in relation to AHI showed that the different criteria for successful treatment were more often met within the therapy group. 40.9% (*n* = 9) of patients receiving Ronch®AP therapy fulfilled Sher criteria, whereas only 20% (*n* = 6) of the control group managed to reduce AHI < 20/h and reach a 50% reduction with respect to baseline AHI. With 31.8% (*n* = 7) the therapy group was significantly more likely to score an AHI < 10 (control group: 6.7%, *n* = 2). The improvement of moderate or severe OSAS to a mild form was over six times more likely for patients under Ronch®AP therapy than for patients receiving no treatment at all (OR = 6.29, 95% CI 1.03–69.61, *p* = 0.027). 18.2% (*n* = 4) of patients under Ronch®AP treatment even managed to lower AHI < 5. In the control group no patients showed an AHI < 5 after 4 weeks (Fig. 5).

Evaluating possible predictors for treatment success, the results showed that the Ronch®AP appliance proved to be more efficient in treating patients with a BMI < 30 kg/m^2^. Their AHI was reduced by − 18.1 ± 12.4/h, whereas patients with a BMI > 30 kg/m^2^ only showed a mean reduction of − 7.3 ± 8.7/h. In addition, all patients reaching an AHI < 5 using Ronch®AP were diagnosed with a BMI < 30 kg/m^2^. Furthermore, positive trends for AHI reduction were especially prevalent in patients suffering from severe OSAS. Compared to an AHI reduction of − 9.6/h in patients with moderate OSAS, patients suffering from severe forms lowered their AHI by − 21.7/h. Tables 5 and 6 give an overview on treatment success in regard to different grades of OSAS severity, BMI and sleeping position.

While acknowledging that Ronch®AP therapy successfully reduced AHI, other sleep parameters showed no statistically significant improvement in comparison with the control group (Tables 3, 4, 5 and 6). Nonetheless the frequency of obstructive apneas and hypopneas was still positively influenced. Apnea index (AI) and hypopnea index (HI) even improved significantly under Ronch®AP therapy when compared with the initial measurements before therapy (*p* = 0.016). However, when compared to the control group no statistically significant improvement was observed (*p* > 0.05). As expected Ronch®AP did not influence central apneas (*d* = − 0.05 ± 4.24). With a difference of 2.03 ± 8.86%, even minimal oxygen saturation showed no significant changes under Ronch®AP (*p* = 0.989). With the initial average oxygen saturation being at 93.31 ± 1.14% and 92.67 ± 1.8% after the 4-week follow-up, Ronch®AP also effected no relevant improvement in comparison with the controls (*p* > 0.05). The amount of respiratory effort-related arousals (RERA) was also assessed. Under Ronch®AP the amount of RERAs was reduced by − 11.62 ± 41.16 events on average. Yet, when compared with a − 0.92 ± 0.09 reduction in the control group the improvement was not significant (*p* = 0.256). Sleep efficacy at baseline substantially differed between both groups. While the therapy group entered the study with a sleep efficacy of 72.81 ± 15.15%, the control group started out with 79.95 ± 9.6%. Under Ronch®AP therapy sleep efficacy improved to 75.76 ± 16.82%, while patients receiving no treatment stabilized at 80.37 ± 8.86%. Another positive trend is evident in the decrease of snoring. The total number of snoring sounds was reduced by − 402.78 ± 956.64 events under Ronch®AP therapy. Accordingly, snoring index decreased by − 72.29 ± 160.25/h from initial 188.47 ± 112.85/h to 116.18 ± 116.27/h. Within the therapy group both parameters significantly decreased when compared with parameter value at baseline (*p* < 0.05). In the control group the reduction of snoring was less prominent (sleep index: *d* = − 13.88 ± 136.49, total amount of snoring sounds: *d* = − 35.20 ± 711.35). Still the changes under Ronch®AP were not significant when compared to the control group (SI: *p* = 0.24, snoring sounds _total_: *p* = 0.192). Sleep profile was analyzed by examining the share of different sleep stages in regard to total sleeping time. On average the share of REM sleep doubled under Ronch®AP therapy compared to patients without treatment (therapy group: 3.30 ± 9.11%, control group: 1.41 ± 6.73%). The share of stage N3 (deep sleep) also minimally increased under treatment (N3: 0.13 ± 6.26%). Proportionally, the share of N1 stage (phase of falling asleep) and N2 stage (light sleep) decreased (N1: − 3.54 ± 12.40%, N2: − 0.52 ± 13.16%). Time of wakefulness after sleep onset (WASO) was measured in minutes. On average, patients receiving Ronch®AP therapy were − 24.72 ± 65.01 min less awake than at baseline. In the control group WASO only decreased by − 5.20 ± 40.91 min. The presented changes in the sleep profile were not statistically significant compared to baseline measurements or to the control group (*p* > 0.05).

**Table 4:** Effect of Ronch®AP therapy in comparison with no treatment: parameter differences *t*(0) − *t*(1)/*t*(0) − *t*(c) after 1 month

Variable	Unit	Ronch®AP *t*(0) − *t*(1)	Control *t*(0) – *t*(c)	Two-sample *t* test	*p* value
AHI	[n/h]	− 16.17 ± 12.40	− 1.95 ± 17.59	*t* = − 14.2, *df* = 49.9	*p* = 0.001
AI	[n/h]	− 7.41 ± 12.60	− 0.97 ± 13.72	*t* = 1.65, *df* = 42.6	*p* = 0.106
HI	[n/h]	− 7.88 ± 10.01	− 1.43 ± 14.67	*t* = 1.77, *df* = 43.5	*p* = 0.084
Apnea obstructiv	[n]	− 31.10 ± 71.46	5.67 ± 85.74	*t* = 1.6, *df* = 44.3	*p* = 0.117
Apnea central	[n]	− 0.05 ± 4.24	− 5.37 ± 20.52	*t* = − 1.31, *df* = 28.9	*p* = 0.201
Apnea total	[n]	− 30.70 ± 72.99	− 1.52 ± 91.44	*t* = 1.22, *df* = 44.7	*p* = 0.23
Hypnea total	[n]	− 28.75 ± 75.10	− 1.96 ± 85.47	*t* = 1.13, *df* = 43.2	*p* = 0.265
SpO2 Ǿ	[%]	− 0.64 ± 1.32	− 0.06 ± 1.55	*t* = 1.38, *df* = 44.9	*p* = 0.174
SpO2 minimal	[%]	2.03 ± 8.68	− 0.64 ± 6.36	*t* = − 1.22, *df* = 37	*p* = 0.232
RERA	[n]	− 11.62 ± 41.16	− 0.92 ± 9.09	*t* = 1.17, *df* = 21.7	*p* = 0.256
Sleep efficiency	[%]	2.95 ± 18.33	0.43 ± 9.78	*t* = − 0.58, *df* = 30.5	*p* = 0.564
N1	[%]	− 3.54 ± 12.40	0.29 ± 11.60	*t* = 1.03, *df* = 37.5	*p* = 0.308
N2	[%]	− 0.52 ± 13.16	− 0.38 ± 13.99	*t* = 0.03, *df* = 40	*p* = 0.973
N3	[%]	0.13 ± 6.26	− 1.24 ± 7.69	*t* = 0.67, *df* = 44.5	*p* = 0.506
REM	[%]	3.30 ± 9.11	1.41 ± 6.73	*t* = − 0.8, *df* = 35.3	*p* = 0.43
WASO	[min]	− 24.72 ± 65.01	− 5.20 ± 40.91	*t* = 1.11, *df* = 27.2	*p* = 0.275
Snoring index	[n/h]	− 72.29 ± 160.25	− 13.88 ± 136.49	*t* = 1.2, *df* = 34.6	*p* = 0.24
Snoring sounds total	[n]	− 402.78 ± 956.64	− 35.20 ± 711.35	*t* = 1.33, *df* = 31.2	*p* = 0.192
ESS	–	− 1.63 ± 3.36	0.63 ± 2.54	*t* = 2.34, *df* = 37.5	*p* = 0.025
PSQI	–	− 1.91 ± 2.31	− 0.10 ± 2.41	*t* = 2.74, *df* = 46.5	*p* = 0.009

**Table 5:** Differences *t*(0) − *t*(1)/*t*(0) − *t*(c) of secondary polysomnographic parameters in regard to randomization group: descriptive analysis and results of two-sample *t* test

Variable	Group	*n*	Mean value ± SD	95% CI		Min	Max	*p* value
				Lower bound	Upper bound			
AI	K	26	− 0.97 ± 13.72	− 6.21	4.26	− 29.6	45.5	0.71
	T	20	− 7.41 ± 12.60	− 13.38	− 1.44	− 39.3	19.0	0.016
HI	K	26	− 1.43 ± 14.67	− 6.52	− 3.65	− 47.4	26.1	0.572
	T	20	− 7.88 ± 10.01	− 13.68	− 2.09	− 27.1	9.6	0.009
Apnea obstructiv Apnea central	K	27	5.67 ± 85.74	− 25.35	36.68	− 181.0	307.0	0.715
	T	20	− 31.10 ± 71.46	− 67.14	− 4.94	− 190.0	163.0	0.089
Apnea total	K	27	− 5.37 ± 20.52	− 11.51	0.77	− 104.0	12.0	0.085
	T	20	− 0.05 ± 4.24	− 7.18	7.08	− 9.0	12.0	0.989
Apnea obstructiv Apnea central	K	27	− 1.52 ± 91.44	− 34.13	31.10	− 195.0	307.0	0.926
	T	20	− 30.70 ± 72.99	− 68.60	7.20	− 190.0	176.0	0.11
Apnea total	K	26	− 1.96 ± 85.47	− 34.04	30.11	− 230.0	191.0	0.902
	T	20	− 28.75 ± 75.10	− 65.33	7.82	− 151.0	161.0	0.12
SpO2 minimal	K	29	− 0.64 ± 6.36	− 3.42	2.14	− 19.7	14.2	0.646
	T	22	2.03 ± 8.68	− 1.16	5.22	− 13.8	32.0	0.206
SpO2 Ǿ	K	26	− 0.06 ± 1.55	− 0.63	0.52	− 3.5	3.9	0.841
	T	21	− 0.64 ± 1.32	− 1.28	0.00	− 3.6	1.9	0.051
RERA	K	24	− 0.92 ± 9.09	− 12.79	10.96	− 31.0	20.0	0.877
	T	21	− 11.62 ± 41.16	− 24.31	1.08	− 185.0	3.0	0.072
Sleep efficiency	K	27	0.43 ± 9.78	− 5.09	5.94	− 28.6	16.0	0.877
	T	22	2.95 ± 18.33	− 3.16	9.07	− 34.4	37.1	0.336
REM	K	28	1.41 ± 6.73	− 1.57	4.39	− 19.1	13.5	0.345
	T	21	3.30 ± 9.11	0.14	6.73	− 17.0	16.7	0.06
N1	K	24	0.29 ± 11.60	− 4.64	5.22	− 35.6	24.3	0.907
	T	19	− 3.54 ± 12.40	− 9.08	2.00	− 35.0	11.6	0.204
N2	K	25	− 0.38 ± 13.99	− 5.88	5.12	− 28.0	37.5	0.89
	T	19	− 0.52 ± 13.16	− 6.83	5.79	− 26.1	26.8	0.869
N3	K	27	− 1.24 ± 7.69	− 4.00	1.52	− 18.0	12.6	0.37
	T	20	0.13 ± 6.26	− 3.08	3.33	− 8.3	15.6	0.938
WASO	K	23	− 5.20 ± 40.91	− 27.46	17.07	− 85.5	122.5	0.64
	T	18	− 24.72 ± 65.01	− 49.89	0.44	− 158.5	114.5	0.054
Snoring index	K	18	− 13.88 ± 136.49	− 85.27	57.50	− 214.6	296.1	0.695
	T	19	− 72.29 ± 160.25	− 141.77	− 2.81	− 399.8	180.3	0.042
Noring sounds total	K	20	− 35.20 ± 711.35	− 414.41	344.01	− 1247.0	1487.0	0.852
	T	18	− 402.78 ± 956.64	− 802.50	− 3.05	− 2060.0	1184.0	0.048

**Table 6:** Response rate of treatment success in regard to different success criteria

Success criteria by group		Severity of OSAS		BMI		Sleep position	
		Moderate (no/yes)	Severe (no/yes)	< 30 kg/m^2^ (no/yes)	≥ 30 kg/m^2^ (no/yes)	Lateral position (no/yes)	Supine position (no/yes)
AHI < 5	Control	17/0 100%/0%	13/0 100%/0%	24/0) 100%/0%	6/0 100%/0%	15/15 50%/50%	28/2 93.3%/6.7%
	RonchAP®	8/2 80%/20%	10/2 83.3%/16.7%	14/4 77.8%/22.2%	4/0 100%/0%	9/13 40.9%/59.1%	20/2 90.9%/9.1%
AHI < 10	Control	16/1 94.1%/5.9%	12/1 92.3%/7.7%	23/1 95.8%/4.2%	5/1 83.3%/16.7%	14/16 46.7%/53.3%	27/3 90%/10%
	RonchAP®	6/4 60%/40%	9/3 75%/25%	12/6 66.7%/33.3%	3/1 75%/25%	6/16 27.3%/72.7%	20/2 90.9%/9.1
Sher-criteria	Control	16/1 94.1%/5.9%	8/5 61.5%/38.5%	18/6 75%/25%	6/0 100%/0%	17/7 70.8%/29.2%	26/2 92.9%/7.1%
	RonchAP®	6/4 60%/40%	7/5 58.3%/41.7%	10/8 55.6%/44.4%	3/1 75%/25%	6/13 31.6%/68.4%	16/6 72.7%/27.3%

**Fig. 6:**
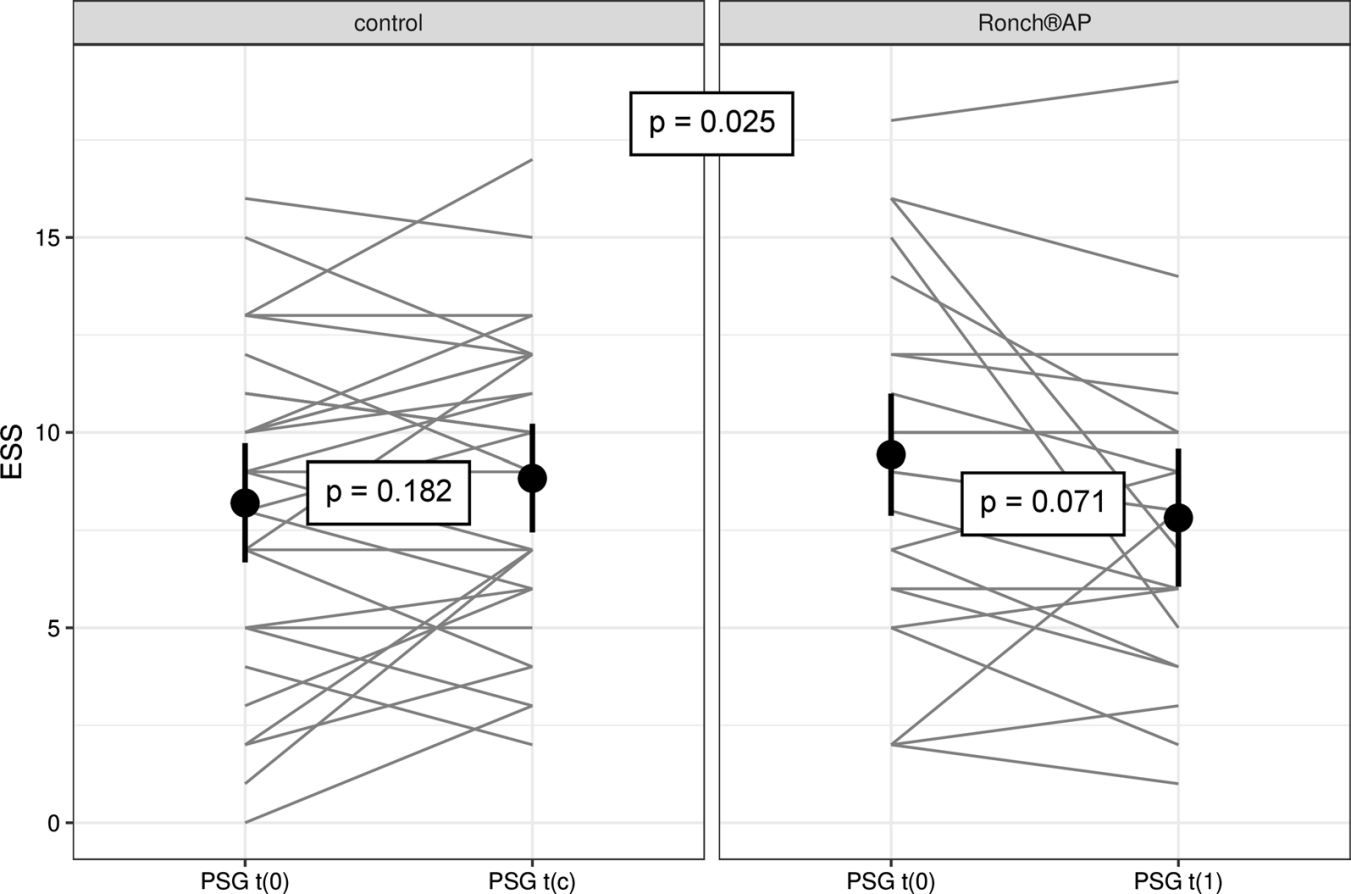
ESS and PSQI

Subjective treatment effectiveness was evaluated by ESS and PSQI. ESS score showed a reduction in daytime sleepiness from initial 9.18 ± 4.73 to 7.82 ± 4.14 under Ronch®AP therapy, whereas the control group showed increased levels of sleepiness (ESS_t(0)_ = 8.2 ± 4.17, ESS_t(c)_ = 8.83 ± 3.8) (Tables 3 and 4, Fig. 6). Even though excessive daytime sleepiness (ECC ≥ 10) was neither observed in the therapy or the control group, mean ESS improvement under Ronch®AP was statistically significant compared to patients with no treatment (*p* = 0.025). According to PSQI sleep quality was also significantly improved compared to the controls (*t* = 2.74, *df* = 46.5, *p* = 0.009). It was lowered from 8.45 ± 3.46 by − 1.91 ± 2.31 to 6.55 ± 3.46. (Tables 3, 4, 5 and 6).

**Fig. 7:**
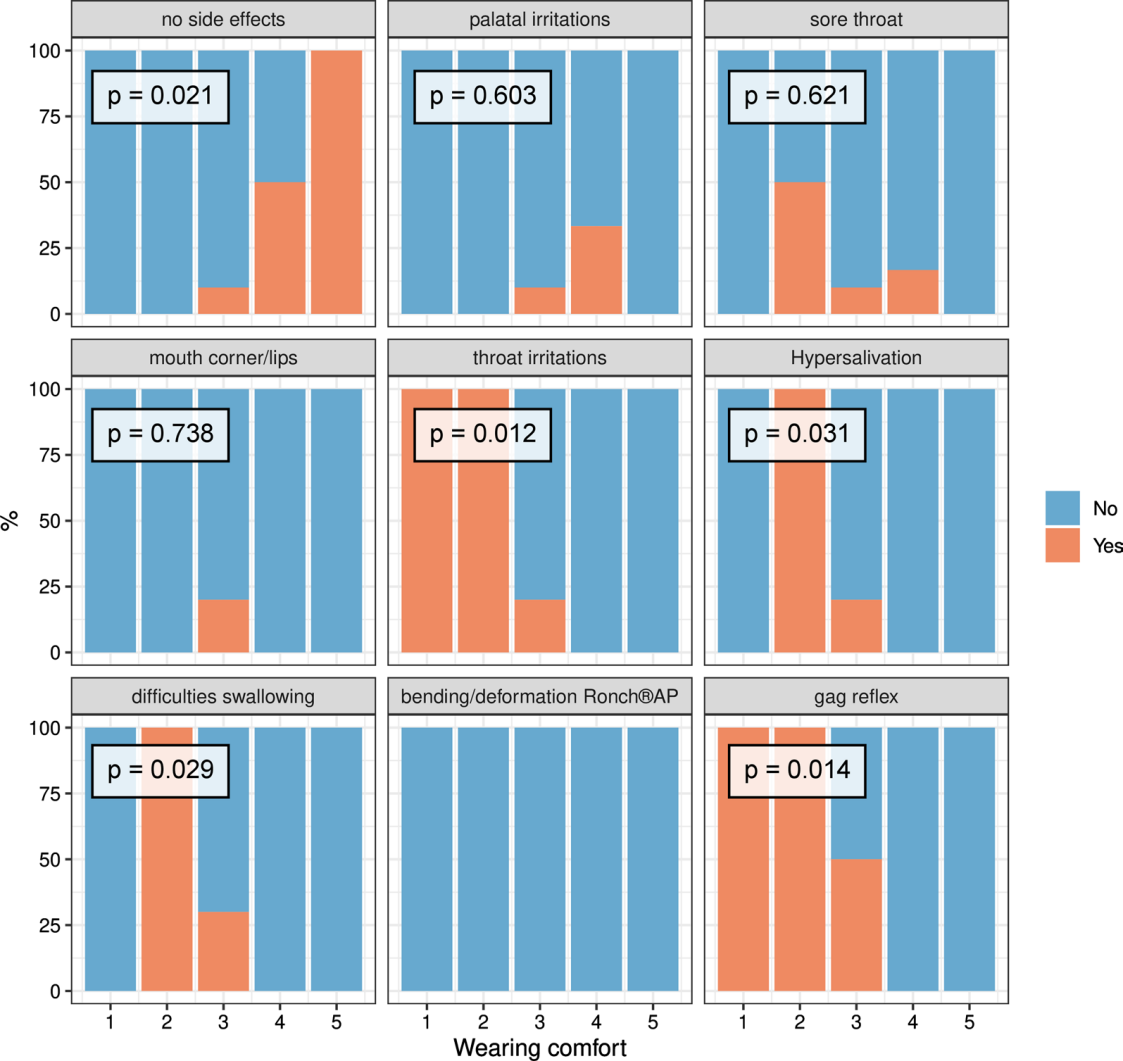
Correlation between side effects and willingness to continue treatment and

22 patients of the therapy group answered the Ronch®AP questionnaire (Tables 5, 6). On average, patients used the device 6.00 ± 1.45 nights per week and 6.14 ± 1.25 h per night. With a mean value of 4.32 ± 1.04, willingness to continue treatment in the future was high. Wearing comfort was only rated mediocre at 3.36 ± 1.00 (Table 7). 25% (*n* = 7) showed no side effects. The most frequently reported side effects included gag reflex (28.57%), throat irritation (17.86%) and difficulties swallowing (17.86%). Table 8 summarizes all side effects and their prevalence. Figure 7 shows how wearing comfort correlated with different side effects. Poor wearing comfort correlated with side effects, such as gag reflex, throat irritations, hypersalivation and difficulties swallowing. High levels of wearing comfort significantly increased the willingness to continue treatment (*χ*^2^ = 34.25, *p* = 0.04).

**Table 7:** Therapy adherence and influential variables of Ronch®AP therapy

Variable	*n*	Mean value ± SD	Min	Max
Willingness of treatment continuation	22	4.32 ± 1.04	1	5
Frequency of usage (nights per week)	22	6.00 ± 1.45	3	7
Time of usage (hours per night)	22	6.14 ± 1.25	3	8
Wearing comfort	22	3.36 ± 1.00	1	5

**Table 8:** Side effects of Ronch®AP therapy

Side effects	*n*	%
Gag reflex	8	28.57
No side effects	7	25.00
Zhroat irritations	5	17.86
Difficulties swallowing	5	17.86
Hypersalivation	4	14.29
Palatal irritations	3	10.71
Sore throat	3	10.71
Irritations of corners of the mouth or lips	2	7.14
Bending/deformation of RonchAP® device	0	0.00

## Discussion

The presented study examined the Ronch®AP palatal device as an alternative conservative treatment option in OSAS therapy. Even though the therapeutic approach to treat OSAS by stenting the retropalatinal space is not novel, little research has been published [21, 22]. So far, no research on Ronch®AP has been published. A similar device is the Velumount® appliance [22]. In contrast to the RonchAP® technique the therapeutic effect relies solely on stenting the velum. Tongue base and the posterior pharyngeal walls are not supported. Therefore, it has to be noted that while comparability of both systems is possible, it is also limited.

The effectiveness of Ronch®AP was examined in a randomized controlled clinical trial over 4 weeks. The study’s strength lies in its design. A controlled, randomized trial minimizes the risk of selection bias. Moreover, cardiorespiratory polysomnography represents the highest diagnostic standard for OSAS and therapeutic management. All polysomnographic testing was conducted in accordance with international standards. The observation interval of 4 weeks was considered suitable for the studies' objective. It allowed the evaluation of the effectiveness of Ronch®AP while upholding ethical standards. As the control group did not receive any treatment during the 4-week time interval, a longer observation period would not have been ethically acceptable.

In comparison with the control collective, AHI was significantly reduced from 35.34 ± 14.9/h to 19.18 ± 14.93/h (*p* = 0.001) under RonchAP® therapy. This compares well to the results of Staub on the Velumount® device after a 6-week trial. He observed a reduction in AHI from 34.6 ± 20.9/h to 19.1 ± 14.2/h [23]. In another clinical cohort trial AHI was even reduced to 13.6 ± 12.2/h [22]. However, with a 24.3 ± 10.1/h baseline AHI was also substantially lower compared to the initial AHI of patients treated with Ronch®AP (AHI_t(0)_ = 35.34 ± 14.9/h).

Analyzing treatment success in regard to different AHI-related success criteria shows that the therapy group performed better in all three categories. By reducing AHI < 10 31.8% patients (*n* = 7) managed to improve their moderate or severe OSAS to mild forms. Considering that the risk of complications increases with the severity of disease, this effect is highly favorable. 40.9% (*n* = 9) of patients receiving Ronch®AP therapy fulfilled Sher criteria. With a response rate of 60% according to Sher criteria Velumount® was more effective [22]. After 4 weeks of regular Ronch®AP usage 4 patients (18.2%) no longer even qualified as OSAS patients when their AHI fell below 5. Positive trends for treatment success were observed for patients with BMI < 30 kg/m^2^ and severe OSAS.

The lack of statistical significance in regard to these predictors might be attributed to the modest size of the examined subgroups.

The statistically relevant improvement of AHI under Ronch®AP compared to the control group was not found in other sleeping parameters. However, measured by their baseline values significant improvements were observed within the therapy group in regard to apnea index and hypopnea index, the total amount of snoring sounds and snoring index. Positive trends transpired in regard to the total amount of obstructive apneas and hypopneas and the amount of respiratory effort-related arousals. In addition, a minor shift in the sleep profile toward a higher share in deep sleep at the expense of light sleep must be positively acknowledged. Patients under Ronch®AP therapy were five times less likely to awaken after sleep onset. In contrast to the Ronch®AP treatment that had no effect on minimal and average oxygen desaturation, De Bruijn observed a significant increase in both parameters under Velumount® [24]. It also must be noted that during follow-up patients receiving no therapy showed better sleep efficacy than patients who were treated with Ronch®AP. Comparability is, however, limited, because both collectives entered the study with substantial differences in sleep efficacy. In addition, it must be recognized that a limiting factor in analyzing secondary sleep parameters was the fact that baseline records were often incomplete. Twenty out of the 60 baseline polysomnographies had previously been conducted alio loco. It is possible that statistically relevant changes in secondary sleep parameters were not detected due to the small sampling size.

The fact that objective improvements (e.g., AHI reduction) are also reflected in patients’ positive subjective assessment of treatment effectiveness is highly beneficial for compliance and therapy adherence. Daytime sleepiness and sleep quality were significantly improved compared to patients without treatment. Considering that daytime sleepiness is one of the main symptoms of OSAS and that patients frequently suffer from poor sleep quality due to frequent awakenings and arousals, these results are encouraging. Different studies on Velumount® therapy support the evidence given in the presented study that palatal oral appliances reduce ESS. This is seen in patients’ positive evaluation of palatal appliances in OSAS therapy [22, 23, 25, 26]. In a questionnaire-based study investigating long-term compliance of Velumount® therapy sleep quality was assessed. Its impact on sleep quality was scaled as ‘clearly better’, ‘better’, ‘equal’, ‘worse’ or ‘clearly worse’. The results showed an improvement in sleep quality by 67%. However, in 6% of cases sleep quality actually deteriorated while using Velumount® due to side effects, such as foreign body sensation and gag reflex [21]. Under Ronch®AP side effects such as gag reflex, throat irritation and difficulties in swallowing were most prevalant. Due to habituation, patients coped rather well with side effects and tolerated them when the patients showed high levels of therapy adherence for the duration of the study. On average, patients used Ronch®AP 6.00 ± 1.45 nights per week and 6.14 ± 1.25 h per night. With a mean time of usage of 6.4 ± 8.1 nights per week and 7.1 ± 0.8 h per night Velumount® therapy was applied slightly more often [22]. However, in regard to initial compliance Ronch®AP performed better. The drop-out rate was estimated at 26.7% (*n* = 8). Accordingly, the compliance after 1 month was 73.3%. Under Velumount® initial compliance was estimated at 56% [25]. While willingness for treatment continuation was promising, wearing comfort was mediocre. This emphasizes the importance of diligent adaptation respecting individual anatomy. Because high levels of wearing comfort significantly increased the willingness to continue treatment, side effects such as gag reflex that minimizes comfortability should be considered during patient selection for future research.

In total, this study’s findings are promising. However, the results should be seen in the light of their limitations. The analysis performed only included patients with moderate and severe OSAS. Even though the results for the presented cohort are significant, the study is not able to give evidence for the effectiveness of Ronch®AP therapy for mild forms of OSAS. Furthermore, in future studies reliability and quality of results could be enhanced by choosing larger patient cohorts and assessing the influence of interesting variables, such as location of obstruction occurrence. Data were reduced by early drop-outs in the adjustment process and by incomplete records. It will be interesting to assess if potential predictors for treatment success are identified in future studies with larger sampling sizes. However, the acknowledgement of drop-outs provides intrinsic information on tolerance capacity of Ronch®AP. As the study presents a first-time scientific evaluation of Ronch®AP, the chosen observation period of 4 weeks surely allowed obtaining important information on principal treatment effectiveness. On the other hand the study was not designed to obtain information on long-term compliance and treatment success.

## Conclusions

The Ronch®AP palatal device is effective in treating moderate and severe OSAS. It significantly reduces AHI as well as daytime sleepiness and significantly improves sleep quality. Patients with a BMI < 30 kg/m^2^ seem to benefit more in comparison with obese patients. As OSAS is a chronic disorder often requiring long-term treatment, a closer evaluation of long-term compliance and stability of treatment success is strongly advised for future studies.

## Data Availability

Data are available by the authors.
